# A Scoping Review of the Role of Artificial Intelligence in Physician Burnout

**DOI:** 10.7759/cureus.88580

**Published:** 2025-07-23

**Authors:** Curtis Ko, Brittany Shectman, Daniel Uy, Cameron Minars, Brian Ingram, Nidhi Chary, Kaitlyn Chung, Graham Girdler, Hoa Le, Sahar Miri, Robin J Jacobs

**Affiliations:** 1 Dr. Kiran C. Patel College of Osteopathic Medicine, Nova Southeastern University, Fort Lauderdale, USA

**Keywords:** ai, burnout, electronic health record, electronic medical record, mental health, work-life balance

## Abstract

Physician burnout is an increasingly prevalent issue affecting many physicians across all specialties and, by proxy, their patient outcomes. One key factor is associated with excessive medical record documentation. Maintaining patient records can be time-consuming, cumbersome, and complicated for some physicians. As such, artificial intelligence (AI) such as ChatGPT or newer programs may provide an effective solution, which may provide reduced human work efforts, including administrative tasks such as documentation. With advances in natural language processing (NLP) tied to AI approaches, the medical field has been open to adopting AI to improve efficiencies tied to documentation and other repetitive clerical duties. This scoping review aimed to examine the impact of AI on physician burnout in the context of medical charting and administrative tasks, excluding its role in diagnosis or patient treatment. Specifically, it aimed to explore existing research to understand the influence of using AI on physician burnout and determine how AI has influenced the experiences of physicians. A thorough search of the peer-reviewed literature, following Preferred Reporting Items for Systematic Reviews and Meta-analyses extension for scoping review (PRISMA-ScR) guidelines, was conducted across three databases (EMBASE, Ovid Medline, and Web of Science) for articles published in English between 2014 and 2024, focusing exclusively on physicians using AI technologies in clinical settings. The initial search yielded 663 articles, which were refined to eight articles after a systemized, rigorous search and selection process. The findings indicate a potential for AI to mitigate physician burnout by decreasing documentation time, improving physician-patient interactions, and enhancing clinical workflow efficiency. Challenges such as overreliance on AI and insufficient familiarity with technology may be tied to misinformation or misuse. These issues highlight areas of further development and research to advance AI technology integration into clinical practice.

## Introduction and background

Physician burnout is an issue growing in incidence and prevalence that negatively affects both healthcare workers and patients, regardless of specialty. Long-term adverse outcomes tied to physician burnout include emotional exhaustion, feelings of inadequacy, and demotivation [1]. In addition, physicians have reported feeling fatigued, irritable, and depressed [2]. The exhaustion faced by physicians not only reflects on their work efforts but can have lasting negative implications on their personal lives. The feelings of inadequacy and depersonalization may stem from substantial stress levels felt by physicians [1,2]. Because burnout is a self-reported syndrome, its prevalence is often underreported, which underscores the need to gain insight into physician burnout and address this unmet need. Physician burnout is frequently assessed with the Maslach Burnout Inventory (MBI) [1], a self-reported survey, which aims to address the feelings of emotional exhaustion, depersonalization, and personal accomplishments. A systematic review found that physician burnout reached a prevalence above 50% in 2014 compared to 45% in 2011, with all U.S. physicians susceptible to developing the condition at some point in their careers [3].

Physician burnout is attributable to both work factors, including a lack of autonomy [1] and electronic health records (EHRs) documentation, as well as individual factors. EHR documentation is associated with excess stress and a decrease in job satisfaction due to increased time spent, which can increase physician exhaustion [2]. Cross-sectional studies have revealed individual factors potentially accounting for an increased susceptibility to feelings of burnout, which include physicians' age, sex, debt incurred from education, and/or partners' occupations [1].

It has also been shown that physician burnout (depending on the specialty) can have negative impacts on workforce attrition and healthcare quality, which can in turn lead to economic burden. Many physicians with symptoms of work fatigue tend to retire early, thus potentially limiting access to healthcare [4]. This decreases the doctor-to-patient ratio, which can have negative consequences (e.g., high turnover), primarily affecting patient care and outcomes. This increase in turnover rate has downstream effects on current physicians, as fewer physicians are left in the workforce, including hospital systems [5]. The cost of replacing a single physician is two to three times the physician's salary, regardless of specialty [5]. Physician burnout is also associated with an increased likelihood of patient safety incidents and patient dissatisfaction [6]. A meta-analysis indicated that burnout doubles the risk of adverse patient incidents, thus affecting patient satisfaction and distrust in physicians [6]. Burnout appears to be most prevalent within hospital settings, among physicians working in emergency/urgent care, radiology, neurology, and general surgery. The prevalence rates of burnout have been reported to be as high as 78% [7]. Psychiatry, oncology, and family medicine have the lowest rates of burnout prevalence at 42.05%, 38.36%, and 35.97%, respectively [7].

Burnout has been shown to disproportionately affect younger physicians, particularly those in the early stages of their careers, with an average age of 34 years [8]. This trend is often attributed to inexperience in managing work-life balance and the high demands of documentation [9,10]. Although younger physicians are generally more receptive to adopting new technologies, the implementation of AI-assisted electronic medical records (EMRs) may yield the greatest benefit for older physicians, who may face greater challenges in adapting to new digital documentation systems and stand to gain the most from technological support.

EHRs include longitudinal electronic documentation used to contain information on patient encounters, store data, and retrieve information. These records have been adopted by most clinical facilities within the United States over the past few decades [11]. Meanwhile, in the United States, before 2009 and the American Recovery and Reinvestment Act, most healthcare facilities were not using EMR systems, primarily because of the expense of implementation [12].

Physicians often face long workdays and heavy patient loads. According to a series of studies based on self-reported information, the average physician sees approximately 20 patients per day, spends approximately 19 minutes with each patient, and spends on average 16 minutes and 14 seconds working on the EMR for each patient [13-15]. Conceptually, if these tasks are performed exclusively, the average physician works approximately 45% of a workday on the EMR. Although the EMR is an excellent tool that helps both healthcare providers and patients in proper healthcare administration, it can also be a major source of physician burnout [16]. One article reported that the interactions of EMR and physicians' interactions with EMRs are a prime example of how an invention has "enslaved us" [17].

AI is broadly defined as a branch of computer science that, through various programs using mathematics and logic, can simulate human intelligence, and in so doing, can perform complex tasks that would otherwise need to be performed by a human, such as understanding language, problem-solving, decision-making, and particularly learning [18]. This particular type of AI addresses language comprehension and falls under the category of natural language processing (NLP) [19]. Within healthcare, the ability of NLPs to assist in clinical administration is particularly evident in the creation, documentation, and transcription of patient interactions [19]. Although it has yet to become the standard in practice, AI has the potential to become the best option for handling highly scrutinized, repetitive tasks such as clinical documentation, whose automation could decrease workloads and patient turnaround times [20].

A large gap lies in the understanding of how burnout is defined. The diagnostic criteria for burnout lack consensus, thus hindering the distinction between burnout and other psychiatric illnesses, such as depression, which share many overlapping symptoms [21-23]. In addition, the exact causes of burnout remain an area of active research. Multiple reasons exist for why one individual might experience burnout, while another might not.

AI in medicine has the potential to decrease documentation time and cognitive burden. Current issues, such as inaccuracy in transcription and translation, must be addressed before AI use can be more universally adopted. Meanwhile, AI may still play a key role in decreasing physician burnout if these issues are addressed through personalization, careful planning, and/or a holistic understanding of AI's strengths and weaknesses. This scoping review involved a preliminary investigation of the MEDLINE database, the Cochrane Database of Systematic Reviews, and the Joanna Briggs Institute (JBI) Evidence Synthesis. To the best of our knowledge, no current or ongoing systematic reviews or scoping reviews on the topic were identified.

Objective and review question

The goal of this scoping review was to examine the implications of integrating AI on physician burnout in the context of medical charting and administrative tasks, excluding its role in diagnosis or patient treatment. The two key questions this scoping review aimed to address included: (1) What existing research has explored the impact of AI use on physician burnout? and (2) How has AI influenced the burdens experienced by physicians? 

## Review

Methods

Search Strategy Details

The electronic databases EMBA SE, Ovid Medline, and Web of Science were used to systematically search articles. The key search terms included: AI, physician, and burnout. Boolean operators such as “AND” and “OR” were used. Each key search term was indexed with an “OR” statement between each synonym and relevant term. For example, “AI” OR “AI”. This procedure was performed for every synonym and relevant keyword. Inclusion criteria for the search included all physicians using AI, articles published in the past 10 years, and publications written in English. Other sources included references relevant to the role of AI in physician burnout that were cited in the articles. The online query search strategy for all three databases (see Appendix, Table 2). 

Inclusion/Exclusion Criteria

The inclusion criteria included studies that were written in English, included physicians (MD/DO) and healthcare facilities that used AI, were published between 2014 and 2024, and involved any geographic location, race, ethnicity, or sex. This review focused exclusively on physicians working with AI-related technology in a medical setting, such as clinical decision support, NLP for charting, ambient documentation tools, or predictive analytics for workflow. The exclusion criteria consisted of non-physician healthcare workers and healthcare facilities not using AI. This study did not explore either patients' use of AI as a replacement for their physicians or physicians' use of AI to help in physician diagnosis and treatment.

Types of Sources

This scoping review considered experimental and quasi-experimental designs, including randomized control trials, non-randomized control trials, cross-sectional studies, and observational studies. Abstracts, case studies, review studies, and poster presentations were excluded due to a lack of necessary detail for comprehensive analysis and evaluation. A preliminary search of MEDLINE, the Cochrane Database of Systematic Reviews, PROSPERO, and JBI Evidence Synthesis was conducted, and no current or ongoing systematic reviews or scoping reviews on the topic were identified to the best of our knowledge. The proposed scoping review was conducted by the JBI method for scoping reviews.

Study/Source of Evidence Selection

Upon completion of the search, all identified citations were combined and uploaded into EndNote (Clarivate, Philadelphia, PA), and the duplicated sources were removed. Titles, abstracts, and the full texts of the selected articles were then screened by two independent reviewers to assess the inclusion criteria for the review. The reasons for the source exclusion of full-text sources not meeting the inclusion criteria were reported. Any disagreements between reviewers at each stage were resolved through discussions, including additional reviewers, as necessary. The results and the inclusion process for the search were reported in full and presented in a Preferred Reporting Items for Systematic Reviews and Meta-analyses extension for scoping review (PRISMA-ScR) flow diagram.

Data Management

Records and data were managed with the use of Rayyan (Rayyan Systems, Inc., Cambridge, MA) and EndNote. Microsoft Excel/Google Sheets (Microsoft Corp., Redmond, WA) were used to organize and visualize the process of delineation and the translation of the search terms for each database.

Data Extraction

Data were extracted from the articles included in the scoping review by two independent reviewers using a standardized form developed collaboratively in Microsoft Excel and Google Sheets. This form recorded key information related to the participants, concepts, contexts, study methods, and key findings associated with the review question. While pilot testing was not conducted, the extraction categories were based on guidance from the JBI Manual for Evidence Synthesis, and both reviewers discussed the form in advance to ensure a shared agreement. Discrepancies during data extraction were resolved through consensus and discussion among the reviewers.

Critical Appraisal of Individual Sources of Evidence 

**Figure 1:**
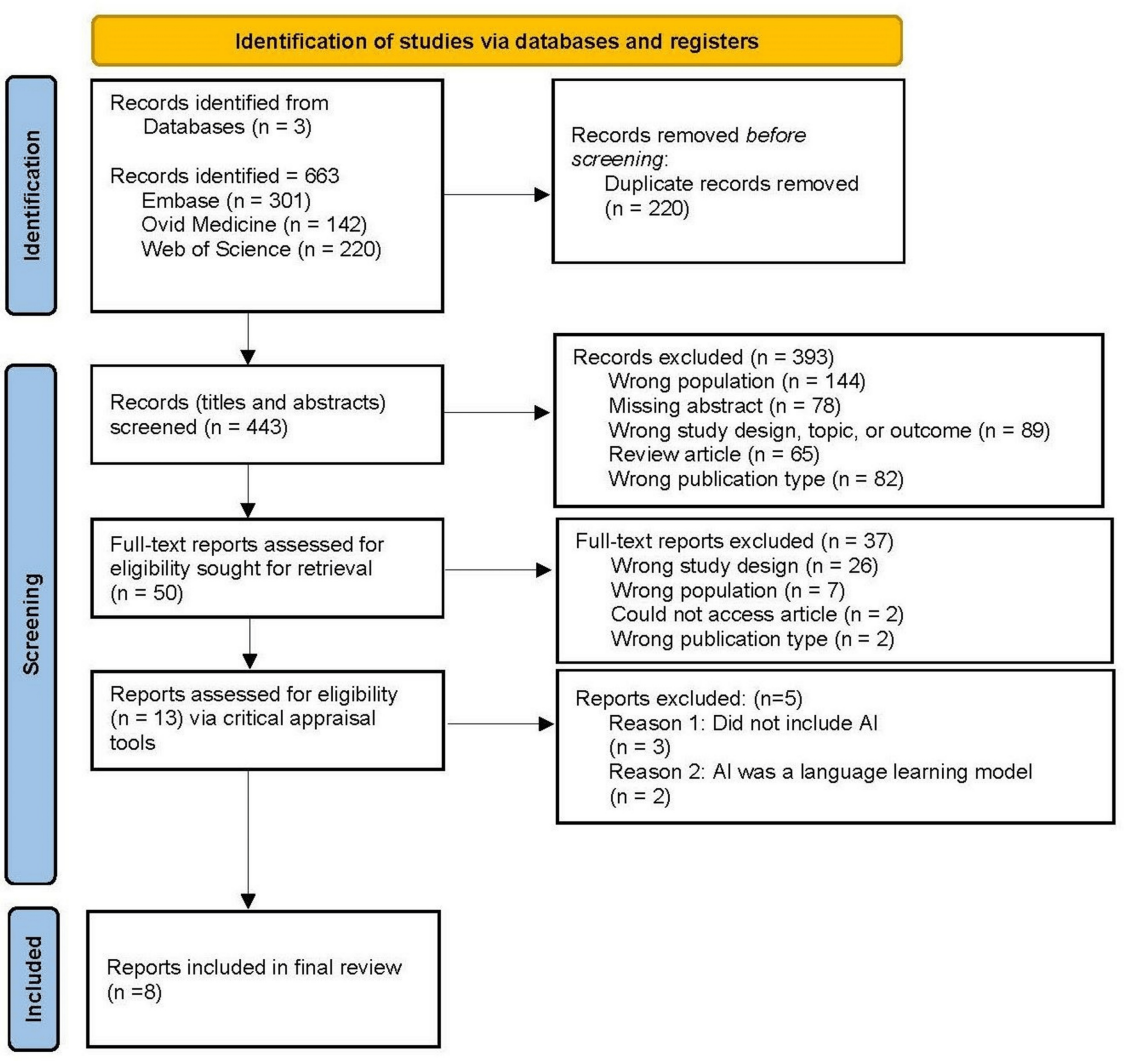
PRISMA flow diagram of the search and selection process. PRISMA: Preferred Reporting Items for Systematic Reviews and Meta-Analyses (PRISMA).

The articles included in the final review were appraised using the appropriate JBI Critical Appraisal tools [24]. Checklists were selected based on the study design. This appraisal determined the quality of the results, relevance, and bias present in the review. The appraisal results were not used to exclude studies but were used to describe the potential limitations and bias within the literature. To ensure rigor, we conducted a systematic and comprehensive evaluation of all initially screened articles by following tier-one screening with the JBI Critical Appraisal Tools, which is a reputable resource for assessing article quality. The JBI checklist carefully addresses research biases, alignment of findings, and essential components, all of which contribute to the overall quality assessment. Articles were categorized by bias risk into three categories: high (scores below 50%), moderate (scores between 50% and 70%), and low (scores above 70%). Two researchers independently reviewed all initially screened articles in depth, each using the appropriate JBI tools to evaluate the content. After the independent assessments, the researchers met to discuss and compare their findings. A consensus was reached through detailed discussions of the relevance and quality of each article. Eight articles were reviewed, all of which met the requirements. Preferred Reporting Items for Systematic Reviews and Meta-Analyses (PRISMA) flowchart of the selection procedure (Figure 1).

Results

Overview

The literature search identified 663 articles. After removal of duplicates, 443 article titles and abstracts were assessed for inclusion. Of the 443 articles screened, 50 articles were assessed for full-text review. Of those 50 articles, 13 were determined to be eligible for assessment via critical appraisal, excluding articles that were not the correct study design, used the wrong patient population, could not be accessed, or were the wrong publication. After critical appraisal, eight of those 13 articles were determined to meet all inclusion criteria and were included in the review. Practicing physicians participated in all studies, and two studies included advanced providers and clinical staff, respectively. All studies explicitly examined the roles of AI in charting, but not diagnosis and treatment. The key factor measured was burnout. Supplemental categories included empathy, information, and efficiency. The consensus was that AI resources, such as ChatGPT, can play a major role in decreasing burnout, but further modifications are necessary to fine-tune AI capabilities.

Among the eight studies selected for the final review, one was conducted in Canada [25]; the rest were conducted in the U.S. Practicing physicians were respondents in all studies, but two studies also included advanced healthcare practitioners, data staff, and administrators [25,26]. The AI modalities used comprised large language models (LLMs) such as ChatGPT (n=5) and language processing technology for transcription (n=2). One study was a survey that assessed the attitudes of primary care physicians and staff without having extensive knowledge of AI [25]. The study methods across studies included quantitative assessments (n=3), qualitative analyses (n=2), observational studies (n=2), and mixed methods (n=1).

AI Use Among Physicians

Utilization of AI among physicians focused primarily on generating automated responses to patient inquiries (n=6). The average response generated by a language learning model was three times the length of a physician's response, and struggled to convey the message that the patient was contacting a provider. LLMs recommended that the patients see a provider and self-referenced their responses [27].

Another reported use of AI was summarizing patient encounters in EMRs (n=2). Misurac and colleagues applied AI in patient-provider visits through the production of a preliminary clinical note by a recording device [26]. The preliminary note was later reviewed by a provider and incorporated into the patient's EMR. Incorporating LLM's into clinical practice has been reported to help decrease providers' documentation burden [28]. The LLM can be used to generate summaries of radiology reports as well as progress notes. The summaries generated were compared with those generated by physicians and evaluated by radiologists and internists to be acceptable.

Reported Burnout Among Physicians

Physician burnout is considered a subjective measure of how an individual responds to professional stressors, thus complicating its assessment. In one study, Owens et al. focused on burnout rates among primary care providers [29]. The study used the Oldenburg Burnout Inventory to assess burnout before the implementation of DAX™ to assist in documentation. The Oldenburg Burnout Inventory was used to assess burnout among two subgroups: disengagement and exhaustion. The scores ranged from 16 to 64, with higher scores indicating greater burnout. Through the use of t-tests and chi-squared tests, this mean score-based data was standardized with linear models. The average score reported was 37.6. Full-time providers reported higher rates of burnout than part-time providers, and the overall amount of burnout among participants was moderate (n=83) [29]. 

In another study, the model used to assess physician burnout was the Stanford Professional Fulfillment Index [26]. Three subcategories assessed using this model were professional fulfillment, work exhaustion, and interpersonal disengagement. Work engagement and interpersonal disengagement were used to determine burnout scores. A score above 3.325 indicated physicians who were actively experiencing burnout. Of the 35 physicians surveyed, the median score was 4.16 before AI implementation and decreased to 3.16, with a p-value of 0.005. In both studies that used two different models, pre-intervention surveys indicated that both groups of providers experienced burnout. 

However, a 2021 survey of 1086 U.S. physician anesthesiologists found that while some respondents were familiar with AI, 48% expressed a favorable attitude toward its use in clinical practice [30]. Respondents cited several motivations for adopting AI in clinical settings, including its potential to improve patient outcomes (81%), reduce healthcare costs (54%), minimize bias (55%), and increase productivity (53%). Interestingly, AI-driven tools, such as NLP and LLMs, provided patients with information taken directly from internet sources without fact-checking for accuracy [31].

Benefits of AI Use for Burnout

Benefits of appropriate AI use in clinical practice have been reported. Most notably, AI used in clinical settings can help with time management. Barak-Corren et al. found that pediatric emergency physicians within a 10-physician cohort spent an average of 3.9 hours on documentation during every shift [32]. Eight out of 10 physicians who elected to incorporate AI into their documentation experienced a 40% decrease in time spent on charting. In addition, Misurac et al. found a statistically significant reduction in self-reported burnout among providers who incorporated this technology into their practice, whose burnout rates decreased from 69% to 43% [26]. Provider feelings of professional fulfillment increased after the implementation of AI in documentation. Furthermore, 28 of the 35 providers reported improved focus on listening to patients during clinical encounters. Owens et al. reported that incorporating AI into documentation decreased the documentation time per encounter by 28.8% and that the time spent documenting outside of scheduled hours also decreased by 11.8% [29]. In addition, providers who used AI in their documentation process were less likely to describe work negatively and were more likely to have energy for leisure activities as well as to feel more engaged and energized at work. The improvement of providers' attitudes and energy levels at work was associated with the effective implementation of AI.

Risks of AI Use by Physicians

A risk of AI usage is that ChatGPT, which was explicitly used as an LLM within four studies, derives its information from the internet and lacks the knowledge of physicians with medical degrees from an accredited institution. Consequently, patients might potentially make incorrect healthcare decisions based on recommendations from AI technology. Baxter et al. used ChatGPT to answer questions within an EMR patient portal [27]. In certain situations, patients who asked for medical advice were told to contact their healthcare providers. Meanwhile, AI also tended to escalate negative patient messages by telling patients to contact the administrative staff for next steps. Participants had a positive outlook regarding AI decreasing burnout but had concerns regarding how AI might affect the doctor-patient relationship and render it impersonal [25].

Studies were grouped in a table by study type (quasi-experimental, qualitative, etc.), year of publication, and methods, thus enabling comparison across studies and the identification of patterns and trends. The articles were then analyzed for key findings, limitations, and purposes, which were added to the final summary table (Table 1). 

**Table 1:** Summary table of the included studies.

Author, Year	Study design	Purpose	Key findings	Limitations
Nash et al., 2023 [25]	Qualitative	To assess the attitudes of primary care physicians and staff in using artificial intelligence (AI) to care for patients.	Primary care physicians and medical staff held generally positive attitudes toward AI, predicting its role in reducing burnout.	Certain healthcare workers may have been underrepresented, and the study was conducted in a community health center, so it is not possible to gauge if the results would translate to other settings.
Misurac et al., 2024 [26]	Quasi-experimental	To explore physician satisfaction with Ambient AI.	Physicians using AI to transcribe patient-clinical conversations, generate preliminary clinical notes for review, and enter notes into EMR showed a significantly reduced physician burnout from 69% to 43%.	The possibility of a type 1 error and the fact that healthcare providers participated on a voluntary basis may not be representative of the population.
Baxter et al., 2024 [27]	Qualitative	This study aims to see if large language models (LLMs) are a feasible way to reduce physician burnout by answering negative patient messages.	LLMs were found to be feasible for drafting patient EHR message responses, aligning with physician communication in relational connection, informational content, and next-step recommendations.	None noted.
Van Veen et al., 2024 [28]	Quasi-experimental	To evaluate whether LLMs could outperform medical experts in clinical text summarization across multiple tasks.	Integrating LLMs into clinical workflows alleviated the documentation burden, allowing clinicians to focus on patient care.	Multiple specialties were not evaluated.
Owens et al., 2023 [29]	Quasi-experimental	To assess the efficacy of ambivalent voice technology, AI, and language processing on primary care physician workload.	AI use in primary care was linked to significantly lower physician burnout, reduced documentation time by 28.8%, and improved job satisfaction.	There was no control group, and longitudinal changes were not seen.
Estrada Alamo et al., 2024 [30]	Cross-sectional	To ascertain the factors that shape anesthesiologists' attitudes toward AI.	Nearly half (48%) of anesthesiologists expressed positive attitudes towards AI in clinical practice.	Lack of comparing American Society of Anesthesiologists respondents to a wider network of anesthesiologists in the United States; differences in demographics; low response rate could lead to biased findings; algorithms can omit jointly significant variables and keep nonsignificant variables that confound each other.
Scott et al., 2024 [31]	Diagnostic test accuracy	To evaluate the quality of Chat-GPT responses to real-world urology messages.	Chat-GPT-generated responses to clinical questions from patients were considered appropriate 50% of the time.	The limitations are that ChatGPT's knowledge base is from the internet. Furthermore, inserting questions into different tabs doesn't allow the software to improve by learning from earlier questions. The last limitation is that this study was used specifically in urology and should be studied in other medical specialties.
Barak-Corren et al., 2024 [32]	Quasi-experimental	To find out if the LLM ChatGPT helps clinicians with documentation while reducing the time required to document.	Pediatric emergency medicine physicians rated Chat-GPT documentation summaries highly for completeness (7.6/10), accuracy (8.6/10), efficiency (8.2/10), and readability (8.7/10). Additionally, Chat-GPT reduced note-taking time by 40% and effort by 33%.	Small sample size, absence of a blind comparison with other summaries, a novelty bias to ChatGPT, and one medical specialty from the same program being evaluated.

Discussion

The results of this scoping review showed ways in which AI can serve to alleviate physician burnout. The articles reported an overall benefit for physicians who experienced increased work stress before the implementation of AI, as well as a decrease in documentation time, increased time spent with patients, and enhanced clinical workflow efficiency. This ultimately decreased burnout experiences among physicians. Although there are many advantages outlined in the research, some studies suggested limitations and even risks of implementing AI within EMR systems.

Perceived Benefits of Electronic Medical Record (EMR) and AI Integration

A correlation was suggested between AI and the alleviation of physician burnout [29]. This finding was associated primarily with decreased administrative burdens tied to AI, which was associated with EMR charting. A major theme identified was that AI tools, such as the integration of ChatGPT, resulted in decreased time spent on documentation and charting tasks [18]. AI embedded within EMR systems can serve to organize a vast amount of patient information at once, thereby enabling physicians to make faster and more accurate diagnoses, potentially streamlining patient care, and increasing both the quality and quantity of care provided.

The findings of this review also suggest that AI has the potential to increase workflow efficacy, particularly through minimizing the time spent on repetitive tasks [29]. Time spent on monotonous tasks, such as scheduling, accounting, and billing, can be decreased with AI-powered programs, which fill in this information for the providers. As such, the number of no-shows and gaps often found in physicians' schedules can be decreased while increasing the time that can be spent with patients. As a result, physicians can focus on listening to their patients without having to worry about assignments, which could easily be completed by AI. AI embedded within an EMR system can also streamline workflow efficiency. By having all a patient’s documentation in one space, AI may be able to help physicians find data faster by returning patient history, laboratory, and imaging findings within seconds. This decreased workload can increase the time available for physicians to spend on non-clinical work and consequently enhance physicians' quality of life. This improved work-life balance, in turn, increases job satisfaction among healthcare providers as well as feelings of personal and professional satisfaction. Although some research has suggested that AI decreases personalized healthcare experiences, many studies suggest that the integration of AI allows physicians to spend less time with the EMRs and more time with patients [32].

AI not only has the potential to increase physicians' quality of life but may also increase documentation quality. AI has been shown to generate concise and patient-friendly summaries from encounter notes. These summaries can help patients understand their care, while removing any medical jargon that a patient might not understand, thus enhancing communication between providers and alleviating patients' stress regarding their visits. AI summaries can help decrease the number of questions that providers receive post-visit. Physicians who used AI-based notetaking during patient interactions found that they were able to focus more on conversations with the patients rather than taking notes [26]. Thus, decreasing the sense of disconnect that patients might feel towards their physicians.

Challenges and Risks of Implementation

Although AI was found to have great potential in reducing physician burnout, there is room for improvement. Various studies included in the review stipulated that AI-driven tools, such as NLP and LLMs, provided patients with information taken directly from internet sources without fact-checking for accuracy [31]. Nash et al. found that a foundation of trust with AI is important to patients in evaluating patient and physician satisfaction with integration [25]. Importantly, the researchers found that most participants were open to using AI, stating that "anything that can help them (providers) and help their client (patients)" would be beneficial to providers [25]. The risk of misinformation provided by AI to patients and physicians can have very dangerous consequences, thus potentially increasing the strain felt by providers who might continually worry about the accuracy of AI-provided information.

Although AI has been shown to decrease physician fatigue and burnout, its general acceptance among healthcare professionals faces several obstacles [9]. The usability of AI and integration into already established practices can place additional stress on physicians and their staff. Without proper training, those who do not use this type of technology might perceive it as an additional burden in their already demanding lives. The lack of standardized AI-EMR training and the cost of implementing such training may pose barriers to the integration of such systems.

Implications for Practice

This review demonstrated the potential ability of AI to decrease physician burnout. Meanwhile, the current lack of validity associated with AI has created a substantial disconnect between physicians wanting to use AI and patients receiving correct information [31]. To maximize the potential of AI integration, future AI-EMR platforms should be able to validate data based on reputable sources. This measure, together with physician and staff training in the accurate use of the platform, could increase the trustworthiness of AI desired by physicians and their patients. Importantly, AI must not be used in place of physicians but should serve as a tool to decrease physicians' stress. By using AI as a tool rather than a replacement for documentation, physicians would gain more time for other tasks, whether personal or work-related.

Implications for Research

Two studies were cross-sectional and did not examine the benefits of AI in alleviating long-term physician burnout [26,30]. Future research should focus on longitudinal studies to evaluate trends in AI use and physician burnout over a longer period. Studies should also examine these trends across various healthcare settings, including urban and rural areas, as well as private and public practices.

Future research should examine specific ways in which AI might help physicians within their specialties, such as electrocardiogram (EKG) analysis for cardiologists or examination of retinal images for ophthalmologists to detect any early signs of a disease. It should also examine personalization features of AI that could be adapted to each physician's preferences. This adaptation would enable physicians to feel more at ease with AI, thus facilitating a smooth transition to AI in EMR systems.

Limitations of the Review Process

As described, research in this field is growing rapidly, and this review describes current findings on this topic. However, biases may have existed given the limited number of databases searched (i.e., three). Possible publication bias might have occurred as all articles included were from the U.S. and Canada (and in English). Furthermore, the results were based only on research published in peer-reviewed journals. Enhancing this study, the inclusion of non-peer-reviewed literature may have offered additional insights or context, addressing gaps not fully covered by peer-reviewed sources.

Limitations of the Studies Included

The limitations of the included studies are grouped into four categories: (1) sample size, (2) timing, (3) setting, and (4) sources of information. The most salient limitation was the sample size (n=7). The experiments conducted were focused on specific medical specialties, and the respondents were recruited voluntarily. As such, although the results supported implementing AI to decrease physician burnout, whether the findings can be generalized across additional specialties is unclear. One study's non-longitudinal design prevented the determination of the role of AI in decreasing physician burnout over time. Other studies used ChatGPT as the LLM, which is derived from the internet rather than an accredited medical institution, thus prompting questions regarding the validity of the information shared with the patients. In another case, only primary care physicians and staff at a community health center were included as participants. Although the results suggested optimism regarding the future of AI, they could not be extended to other healthcare settings. 

## Conclusions

This scoping review aimed to examine the impact of AI on physician burnout in the context of medical charting and administrative tasks, excluding its role in diagnosis or patient treatment. While the primary focus was on non-clinical duties, we acknowledge that physician burnout is multifactorial, and aspects of patient care may also play a contributory role. Specifically, we aimed to explore existing research to understand the impact of AI use on physician burnout and determine how AI has influenced the stressors and burdens experienced by physicians. Results of this review indicated that AI was an effective method to decrease physician burnout by increasing workflow efficiency and decreasing administrative burdens, as evidenced by eight studies reporting evidence supporting this claim. The findings indicated that AI could help diminish physician burnout by conducting tasks, such as summarizing documents and decreasing the time spent on charting. Meanwhile, adverse outcomes tied to AI use within EMRs include a lack of credibility, which is partly attributable to some of the LLMs obtaining information directly from the internet, without verification of validity or accuracy. One limitation of this scoping review is the small sample sizes of the included studies. Future research should focus on conducting longitudinal studies to assess the long-term effects of AI on physician burnout. In addition, by refining AI models to include specialty-specific needs, healthcare systems could use these valuable AI tools to improve patient care outcomes and eradicate physician burnout.

## Appendices

**Table 2:** Search queries for Embase, Medline, and Web of Science.

No.	Search query Embase	Results
#8	#3 AND #6 AND (english)/lim AND (abstracts)/lim AND (2014-2024)/py	187
#7	#3 AND #6	301
#6	#4 OR #5	1,36,963
#5	artificial intelligence':ti,ab,kw OR 'ambient intelligence':ti,ab,kw OR 'artificial general intelligence':ti,ab,kw OR 'automated reasoning':ti,ab,kw OR 'cognitive technology':ti,ab,kw OR 'computer heuristics':ti,ab,kw OR 'explainable artificial intelligence':ti,ab,kw OR 'generative artificial intelligence':ti,ab,kw OR 'multicriteria decision analysis':ti,ab,kw OR 'responsible artificial intelligence':ti,ab,kw OR 'ai':ti,ab,kw	1,30,208
#4	artificial intelligence'/exp	1,13,999
#3	#1 OR #2	29,555
#2	burnout, professional':ab,ti,kw OR 'career burn-out':ab,ti,kw OR 'career burnout':ab,ti,kw OR 'occupational burn-out':ab,ti,kw OR 'occupational burnout':ab,ti,kw OR 'professional burn-out':ab,ti,kw OR 'professional burnout':ab,ti,kw OR 'burnout':ab,ti,kw OR 'burn-out':ti,ab,kw OR 'burn-out syndrome':ti,ab,kw OR 'burnout syndrome':ti,ab,kw OR 'burnout, psychological':ti,ab,kw OR 'psychological burn-out':ti,ab,kw OR 'psychological burnout':ti,ab,kw OR 'burnout':ti,ab,kw	30,364
#1	burnout'/exp	33,860

No.	Search query Medline	Results
#8	limit 7 to (abstracts and english language and yr="2014 -Current")	127
#7	5 and 6	142
#6	3 or 4	272,133
#5	1 or 2	30,327
#4	('artificial intelligence' or 'ambient intelligence' or 'artificial general intelligence' or 'automated reasoning' or 'cognitive technology' or 'computer heuristics' or 'explainable artificial intelligence' or 'generative artificial intelligence' or 'multicriteria decision analysis' or 'responsible artificial intelligence' or 'ai').ab,ti,kf.	96,978
#3	exp Artificial Intelligence/	208,243
#2	('burnout, professional' or 'career burn-out' or 'career burnout' or 'occupational burn-out' or 'occupational burnout' or 'professional burn-out' or 'professional burnout' or 'burnout' or 'burn-out' or 'burn-out syndrome' or 'burnout syndrome' or 'burnout, psychological' or 'psychological burn-out' or 'psychological burnout' or 'burnout').ab,ti,kf.	24,673
#1	exp Burnout, Professional/	18,557

#	Search query Web of Science	Results
5	#3 AND #4	51
4	TS=("andrologist" OR "anesthesiologist" OR "cardiologist" OR "dermatologist" OR "diabetologist" OR "emergency physician" OR "endocrinologist" OR "epileptologist" OR "female physician" OR "foreign physician" OR "gastroenterologist" OR "general practitioner" OR "geriatrician" OR "gerontologist" OR "gynecologist" OR "hematologist" OR "hepatologist" OR "hospital physician" OR "immunologist" OR "infectious disease specialist" OR "intensivist" OR "internist" OR "medical geneticist" OR "neonatologist" OR "nephrologist" OR "neurologist" OR "obstetrician" OR "occupational physician" OR "oncologist" OR "ophthalmologist" OR "orthopedic specialist" OR "osteopathic physician" OR "otolaryngologist" OR "pathologist" OR "pediatrician" OR "phlebologist" OR "physiatrist" OR "podiatrist" OR "psychiatrist" OR "pulmonologist" OR "radiologist" OR "rheumatologist" OR "surgeon" OR "urologist" OR "vaccinologist" OR "venereologist" OR "named groups of persons" OR "named groups by occupation" OR "health care personnel" OR "medical personnel" OR "physician" OR "andrologist" OR "anesthesiologist" OR "cardiologist" OR "dermatologist" OR "diabetologist" OR "emergency physician" OR "endocrinologist" OR "epileptologist" OR "female physician" OR "foreign physician" OR "gastroenterologist" OR "general practitioner" OR "geriatrician" OR "gerontologist" OR "gynecologist" OR "hematologist" OR "hepatologist" OR "hospital physician" OR "immunologist" OR "infectious disease specialist" OR "intensivist" OR "internist" OR "medical geneticist" OR "neonatologist" OR "nephrologist" OR "neurologist" OR "obstetrician" OR "occupational physician" OR "oncologist" OR "ophthalmologist" OR "orthopedic specialist" OR "osteopathic physician" OR "otolaryngologist" OR "pathologist" OR "pediatrician" OR "phlebologist" OR "physiatrist" OR "podiatrist" OR "psychiatrist" OR "pulmonologist" OR "radiologist" OR "rheumatologist" OR "surgeon" OR "urologist" OR "vaccinologist" OR "venereologist")	4,47,130
3	#1 AND #2	220
2	TS=("burnout, professional" or "career burn-out" or "career burnout" or "occupational burn-out" or "occupational burnout" or "professional burn-out" or "professional burnout" or "burnout" or "burn-out" or "burn-out syndrome" or "burnout syndrome" or "burnout, psychological" or "psychological burn-out" or "psychological burnout" or "burnout")	53,343
1	TS=("artificial intelligence" or "ambient intelligence" or "artificial general intelligence" or "automated reasoning" or "cognitive technology" or "computer heuristics" or "explainable artificial intelligence" or "generative artificial intelligence" or "multicriteria decision analysis" or "responsible artificial intelligence" or "ai")	2,84,087
